# A Gravity-Triggered Liquid Metal Patch Antenna with Reconfigurable Frequency

**DOI:** 10.3390/mi12060701

**Published:** 2021-06-16

**Authors:** Peng Qin, Guan-Long Huang, Jia-Jun Liang, Qian-Yu Wang, Jun-Heng Fu, Xi-Yu Zhu, Tian-Ying Liu, Lin Gui, Jing Liu, Zhong-Shan Deng

**Affiliations:** 1 CAS Key Laboratory of Cryogenics, Technical Institute of Physics and Chemistry, Chinese Academy of Sciences, Beijing 100190, China; qinpeng17@mails.ucas.edu.cn (P.Q.); wangqianyu19@mails.ucas.edu.cn (Q.-Y.W.); fujunheng17@mails.ucas.edu.cn (J.-H.F.); liutianying17@mails.ucas.edu.cn (T.-Y.L.); lingui@mail.ipc.ac.cn (L.G.); jliu@mail.ipc.ac.cn (J.L.); 2 School of Future Technology, University of Chinese Academy of Sciences, Beijing 100049, China; 3 School of AI—Guangdong & Taiwan, Foshan University, Foshan 528225, China; hgl@fosu.edu.cn; 4 School of Physics and Telecommunication Engineering, Yulin Normal University, Yulin 537000, China; shuigpjd@163.com; 5 Department of Biomedical Engineering, School of Medicine, Tsinghua University, Beijing 100084, China; zxy19@mails.tsinghua.edu.cn

**Keywords:** EGaIn, frequency reconfigurable, gravity field, liquid metal, patch antenna, stereolithography

## Abstract

In this paper, a gravity-triggered liquid metal microstrip patch antenna with reconfigurable frequency is proposed with experimental verification. In this work, the substrate of the antenna is quickly obtained through three-dimensional (3D) printing technology. Non-toxic EGaIn alloy is filled into the resin substrate as a radiation patch, and the NaOH solution is used to remove the oxide film of EGaIn. In this configuration, the liquid metal inside the antenna can be flexibly flowed and deformed with different rotation angles due to the gravity to realize different working states. To validate the conception, the reflection coefficients and radiation patterns of the prototyped antenna are then measured, from which it can be observed that the measured results closely follow the simulations. The antenna can obtain a wide operating bandwidth of 3.69–4.95 GHz, which coverage over a range of frequencies suitable for various channels of the 5th generation (5G) mobile networks. The principle of gravitational driving can be applied to the design of reconfigurable antennas for other types of liquid metals.

## 1. Introduction

In recent years, the demand for communication capabilities has been significantly increased. Therefore, there have been many research advances in the design and implementation of multi-band, ultra-wideband, and reconfigurable antenna technologies [1]. Compared with the multi-band and ultra-wideband antennas, at any given instant of time, the frequency reconfigurable antennas only work in a part of the entire working frequency band. Therefore, this kind of antenna has a superior noise suppression effect on the unused frequency bands.

In common solid-state circuits, switching elements such as radio frequency microelectromechanical systems (RF-MEMS) [2], p-type intrinsic n-type (PIN) diodes [3], and varactor diodes [4], are often used to achieve antenna reconfiguration capability. The efficiency of these reconfigurable antennas may be limited by the application of too many semiconductor devices, which may also cause nonlinear problems. In liquid antennas (e.g., water [5], liquid crystal [6], transformer oil [7], organic solvents [8], and liquid metal [9]), the fluidity and deformability of the fluid are employed to realize changes in the antenna geometry and its radiation characteristics. The usage of these liquid antennas has reduced the number of semiconductor switches.

For instance, in [5], a very high frequency (VHF) seawater monopole antenna was presented for maritime wireless communications. The center frequency of this antenna can be varied between 62.5–180.2 MHz. Liquids such as water and ethyl acetate are economical, but they have a high dielectric loss at higher operational frequencies. In [6], a liquid crystal-based frequency tunable circular ring patch antenna is designed, which achieves a tuning range of 2.427–2.532 GHz but with a gain of 0.1 dBi. To reduce losses, adopting a low-loss transformer oil has been suggested in [7], where a frequency reconfigurable patch antenna was designed to achieve a tuning range from 1.42 GHz to 1.96 GHz.

Liquid metals have also attracted widespread attention in designing reconfigurable antennas due to their rheological and metallic properties [10]. The deformation and actuating strategies of the liquid metal often have implications on the design, the implementation process, and the actual dimension of the reconfigurable antenna.

The existing techniques of deforming and reconstructing liquid metal can be divided into mechanical and electric actuating methods. Mechanical actuating includes, e.g., methods such as pressurization by a syringe [11,12,13,14] or a pump [15,16,17], thermal expansion [18], gravity [19,20], or external forces [21,22,23]. Instances of electric actuating include electrochemistry [24,25] and capillary electrowetting [26,27]. There are advantages and disadvantages in those driving modes of the liquid metal. External forces [18,19,20] or syringe injection [9,10,11,12] methods may not be convenient enough. Pumping methods [13,14,15] often require specially designed auxiliary flow channels. In contrast, it is more convenient to use electrochemical [24,25] and capillary electrowetting [26,27] driving methods. However, due to the introduction of electrochemical reactions, the liquid metal and solution would continue to be consumed. The reconfigurable antenna designed based on the thermal expansion principle of liquid metal can achieve non-contact control [16], but it takes a long time to stabilize the temperature and requires relatively large heating power consumption.

Under the action of gravity, the liquid flows upon the adjustment of the antenna’s position. This phenomenon seems to be an issue that should be avoided when designing a liquid antenna. However, it may be also considered as a simple, yet effective, reconfiguration method. For example, in [19] mercury was used to design a component that is equivalent to a single-pole double-throw switch. For two different angles of +90° and −90°, the component connects different parts, realizing two working states of the antenna. However, considering the toxicity of mercury and the complexity of its processing, the researchers ended up using copper instead of mercury.

In practice, mercury was the earliest liquid metal used in antenna design [17,19,28]. Currently there is a global trend to restrict using mercury [29]. Instead, gallium, gallium-based alloys, and bismuth-based alloys are considered beneficial alternatives to mercury due to their low volatility and toxicity [27,30]. The melting points of gallium and common bismuth-based alloys are higher than room temperature (25 °C), so elemental gallium and bismuth-based alloys are not suitable for applications that need to remain in a liquid state at room temperature. Therefore, in this work, EGaIn (a kind of gallium-based alloy, with a melting point of 15.5 °C [18]) was selected from many types of liquid metals. However, compared with mercury, EGaIn is more prone to oxidation [17]. Under the action of a small amount of oxygen, a thin and dense oxide layer is formed on the surface of EGaIn and can prevent further oxidation [31]. Additionally, this oxide layer will increase adhesion and affect the smooth flow of EGaIn [32]. Therefore, in many studies [12,24,25,26,27], acidic or alkaline electrolytes are used as a highly effective way to remove the oxide film of EGaIn. Here, for the same reason, a NaOH solution is used to fill a part of the patch.

The patch antennas with a simple structure are widely used in integrated circuits, which is due to their convenient integration and cost-effectiveness. By applying the principle of liquid metal deformation under gravity, in this paper, a frequency reconfigurable microstrip antenna is proposed for the first time. The three-dimensional (3D) printing technology is then applied to fabricate the substrate in an integrated and fast approach, and the cavity of the substrate is filled with EGaIn. The antenna is rotated along the axis of the patch, resulting in the flow and deformation of the liquid metal inside the cavity. For different rotation angles, the antenna is changed to various patch shapes, and its radiation characteristics, therefore, are changed. Within a certain range of −30° to +30°, the rotation angle shows an obvious functional relationship with the resonance frequency. Therefore, the angle of rotation of the antenna could also be calculated based on the measured resonance frequency.

In addition to being a broadband reconfigurable antenna, the proposed patch antenna is a promising candidate for detecting the inclination in practical scenarios such as construction, transportation, logistics, aerospace, and robotics.

## 2. Antenna Design

**Figure 1:**
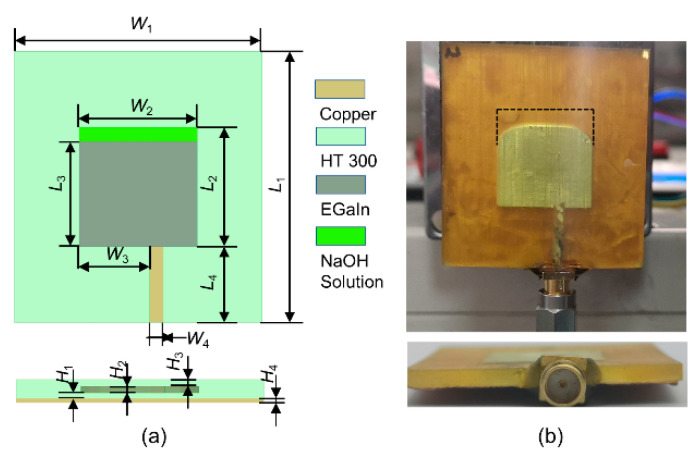
The geometry of the proposed antenna: (**a**) schematic diagram; (**b**) physical diagram.

To verify the feasibility of the proposed reconstruction strategy, a simplified liquid metal patch antenna is designed. The antenna is expected to work at 4–5 GHz. It is configured in a rectangular patch structure, which is illustrated in Figure 1. The antenna comprises of the radiation patch, the substrate, the microstrip line, the subminiature version A (SMA) connector, and a ground plane.

The radiation patch is made of EGaIn (which is a liquid metal with the composition of 75.5% gallium and 24.5% indium, and with an electrical conductivity of σ = 3.46 × 10^6^ S/m [33]), and the microstrip line is made of copper (with an electrical conductivity of σ = 5.96 × 10^7^ S/m [34]). The positions of the radiation patch and microstrip line are reserved while processing the substrate. The substrate is fabricated by 3D printing with photosensitive resin. To ensure that the hollow structure does not collapse, the radiation patch is covered with a dielectric material with a thickness of 1 mm (*H*_1_ and *H*_3_), and the thickness of the cavity at the center *H*_2_ (i.e., the thickness of the radiation patch and the microstrip line) is set to 0.5 mm. Note that if *H*_1_ and *H*_3_ are too small, the covering layer might be easily collapsed (as shown in Figure S1). The initial value of the patch length *L*_3_ can be estimated by the following equation [35]:

(1) $$L_{3}\approx \frac{c_{0}}{2f_{c}\sqrt{\varepsilon_{r}}}$$

where *f_c_* is the center frequency that is expected to be 4.5 GHz, *c*_0_ is the speed of light in the vacuum, and *ε*_r_ is the relative dielectric constant. The substrate material is *High Temperature^®^ 300* (*HT^®^ 300*), which is a high-temperature resistant resin. It should be pointed out that *Figure*^®^ *4* is the name of the 3D printer that can process *HT^®^ 300*. The *Figure*^®^ *4* printer and the *HT^®^ 300* resin are both produced by 3D Systems Corporation, Wilsonville, OR, USA.

**Figure 2:**
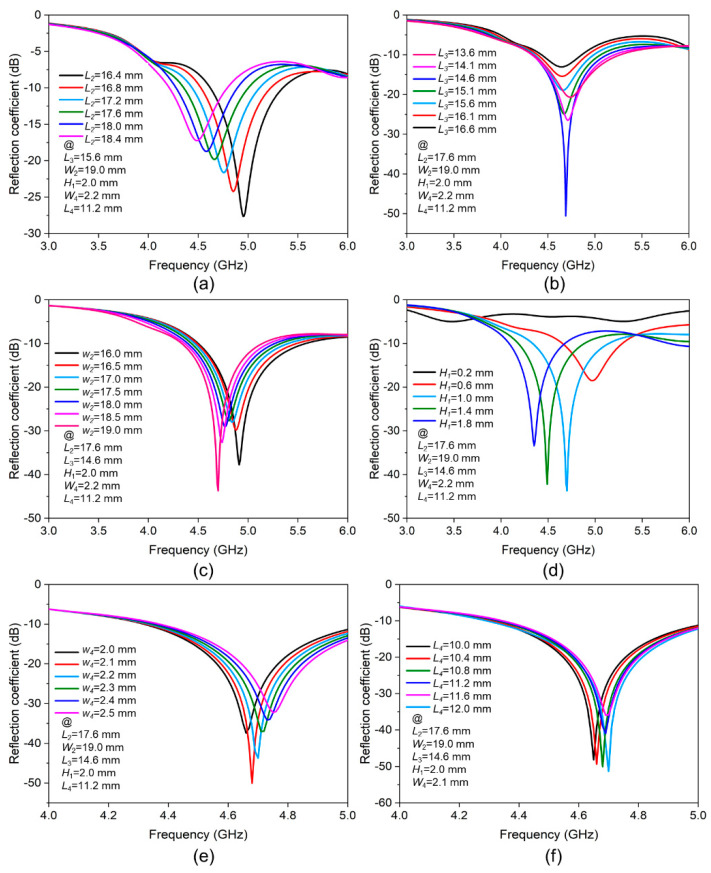
The impact of geometric parameters on the reflection coefficients of the antenna: (**a**) *L*_2_, (**b**) *L*_3_, (**c**) *W*_2_, (**d**) *H*_1_, (**e**) *W*_4_, and (**f**) *L*_4_.

The measured dielectric constant *ε*_r_ of *HT^®^ 300* is 2.7, and the dielectric loss tangent tan*δ* is 0.01 at 4.5 GHz. The antenna works at its dominant mode TM01. The initial values *L*_4_ (11.2 mm) and *W*_4_ (2.3 mm) of the microstrip line are exported by the transmission line calculator Txline. The obtained geometric structure is then modeled and optimized in the full-wave electromagnetic high-frequency structure simulator (HFSS). A part of the optimization process is shown in Figure 2. Figure 2a describes the impact of *L*_2_ on the antenna impedance matching. Increasing *L*_2_ can reduce the resonance frequency, but its reflection coefficient also increases, so the intermediate value of 17.6 mm is selected. Figure 2b shows that the impedance matching is best when *L*_3_ is equal to 14.6 mm. It can be seen from Figure 2c that when *W*_2_ increases and is closed to the value of *L*_2_, the impedance matching becomes worse. However, when *W*_2_ continues to increase, the impedance matching is improved again, so *W*_2_ is set to 19.0 mm. Figure 2d illustrates the influence of the thickness of the dielectric layer between the liquid metal and the ground plane (*H*_1_) on the impedance matching of the antenna. Regardless of whether the thickness *H*_1_ is too large or too small, it is not conducive to the antenna, so an optimized value of 1.0 mm is selected. Figure 2e,f show the results of optimizing the width *W*_4_ and length *L*_4_ of the microstrip line, and they are set to 2.1 mm and 12 mm, respectively. All the optimal dimensions are presented in Table 1.

## 3. Fabrication Process

### 3.1. D printing

A sufficient amount of gallium and indium are weighed according to the mass ratio of 74.5:24.5, and the EGaIn alloy is obtained by smelting in a vacuum melting furnace (Shanghai Mengting Instrument Co., Shanghai, China) at 200 °C for 2 h.

**Figure 3:**
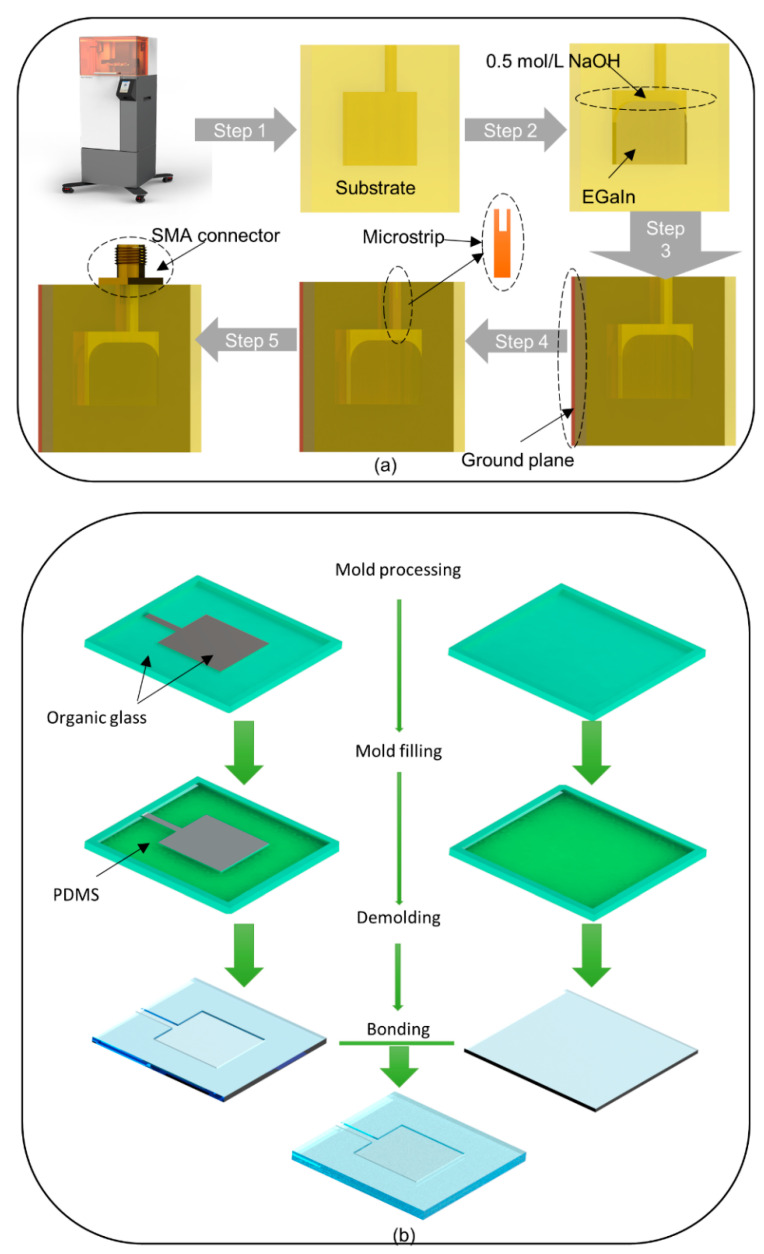
(**a**) Process flow diagram of the antenna manufacturing based on 3D printing. (**b**) Part of the antenna manufacturing process based on molds. This thin patch structure is a part of the mold also made of organic glass, and it is treated as gray for clear display.

The process that is followed after smelting the metal is shown in Figure 3a. Compared with lithography and mechanical processing, 3D printing is a much simpler technique. Stereolithography (SLA), a high-precision 3D printing technology, is used to process the substrate. As mentioned earlier, when testing the dielectric constant of the resin, the resin used in this study is *HT^®^ 300*. This is because the heat-resistant temperature of *HT^®^ 300* can reach up to 300 °C, hence it meets the requirement for temperature-resistant performance. In fact, the original materials and the corresponding printers used are *Accura^®^ 60* photosensitive resin and *ProX^®^ 800*, both produced by 3D Systems Corporation, Wilsonville, OR, U.S. However, the *Accura^®^ 60* photosensitive resin undergoes bending deformation after being exposed to high temperature (see Figure S2a), which would change the antenna structure. The performance of these two materials (*Accura^®^ 60* and *HT^®^ 300*) are compared in Table S1. The actual substrates processed by the two photosensitive resins are also shown in Figure S2b.

Our calculations show that 0.3 mL of EGaIn alloy is required for the corresponding geometric dimensions shown in Figure 1. The position of the microstrip line is used as an injection hole and the alloy is first injected into the cavity with a syringe. Then, 0.5 mol/L NaOH solution (with the dielectric constant *ε*_r_ of 69.93, and the dielectric loss tangent tan*δ* of 0.79 at 4.5 GHz [36]) is injected to fill the remaining cavity. The microstrip line is then cut into a notch (see the illustration in Figure 3a) to place the inner conductor of the SMA connector. This is because the SMA inner conductor and the copper microstrip line can be soldered and connected.

Some copper and tin oxides may be generated in the wire cutting and soldering processes. Therefore, sandpaper is used to carefully polish the surface of the microstrip line until it is smooth. Here, copper is used as the microstrip line instead of continuing to be filled with liquid metal. This is because copper is easier to encapsulate to ensure the sealing of the antenna. A copper foil with a thickness of 0.1 mm is then stuck on the bottom surface of the substrate as a ground plane. The microstrip line is then inserted into the reserved position (the injection port mentioned above), and the outer conductor of the SMA connector is soldered to the ground plane.

During the soldering process of the outer conductor of the SMA connector and the ground plane, the high temperature is conducted to the substrate. This is, in fact, the same heat source that deformed the substrate that was made of *Accura*^®^ 60 resin. Finally, a small amount of glue is applied to the interface of the SMA connector, the microstrip line, and the substrate to further improve the sealing performance and ensure position stability. Figure 1b displays the image of the actual antenna prepared by the above process.

### 3.2. Molding

The molding (see Figure 3b) has also been used when exploring the processing technology of the substrate. Polydimethylsiloxane (PDMS) is used as the substrate material. The model number of PDMS is SYLGARD™ 184, which is produced by Dow Corning, Midland, MI, U.S. First, the base and curing agent of PDMS are weighed at a weight ratio of 10:1 and stirred for 30 min until fully mixed. Then, the PDMS is kept under vacuum for 30 min to remove air bubbles generated during the stirring process. After that, PDMS is poured into the molds made of organic glass, and then the molds are placed on a heating platform at 65 °C for 3 h to fully cure the PDMS. Subsequently, two films made of the cured PDMS with antenna structure are peeled off the molds. Finally, an ion bonding machine (model number is YZD08-2C, Yanzhao Technology, Tangshan, China) is used to treat the two surfaces of the two films, where the surface of the one film has a concave patch structure, and the surface of the other film is smooth. Additionally, then the two surfaces are bonded to obtain a complete patch antenna substrate (see Figure S3).

Through the above multiple steps, the substrate is processed, and the 3D printer is only needed in one step. The subsequent steps of the antenna manufacturing are the same as those used in the 3D printing method. Antennas with the substrate made of PDMS are flexible and can conform to some simple curved surfaces. There are, however, some problems because of their flexibility. For instance, the structure of the antenna is changed if it is connected to the cable during the measuring. In comparison, the 3D printing method is simpler and cheaper. In the end, the process route of the 3D printing is selected.

## 4. Measurement of the Contact Angle

After the proposed antenna was manufactured, it is noticed that the walls of the liquid metal and the resin material are not set at the right angle, but there is a certain arc (see Figure 1). This is because the liquid metal was not wetting the surface of the resin completely based on the NaOH solution. The quality of the wetting can be characterized by the contact angle. The smaller the contact angle is, the better the wetting effect would be. For example, if the contact angle is 0°, it means complete wetting; if the contact angle ranges between 0° and 90°, it means incomplete wetting; if the contact angle varies between 90° and 180°, it means no wetting; and, if the contact angle is equal to 180°, it means no wetting at all.

Liquid metal and NaOH solution are poured into the resin cavity, and after reaching an equilibrium state, the relationship between the contact angle and the surface tensions follows the following equation:

(2) $$\gamma_{\mathrm{Resin}-\mathrm{NaOH}}=\gamma_{\mathrm{Resin}-\mathrm{EGaIn}}+\gamma_{\mathrm{Resin}-\mathrm{NaOH}}\times \theta_{w}$$

where γ_Resin-NaOH_ is the surface tension between the resin and NaOH solution, γ_Resin-EGaIn_ denotes the surface tension between the resin and EGaIn, γ_EGaIn-NaOH_ is the surface tension between EGaIn and NaOH solution, and *θ_w_* is the contact angle between the liquid metal and the resin surface.

**Figure 4:**
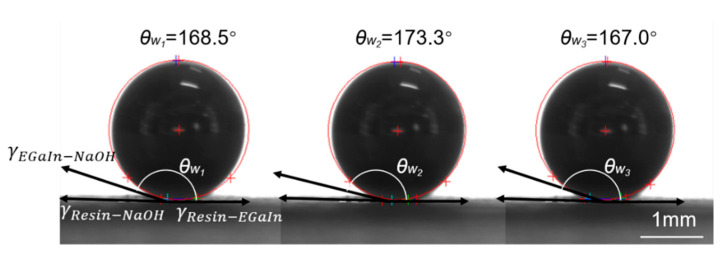
Three measurement results of the contact angle in 0.5 mol/L NaOH solution.

In the 0.5 mol/L NaOH solution, the contact angle, *θ_w_*, is measured three times and shown in Figure 4. The five-point fitting method is then used to obtain the contact angle. Finally, the average value of the three measurements, 169.6°, is taken as the contact angle. The contact angle *θ_w_* is, however, very large since the liquid metal and resin are very non-wetting. The simulation model could be approximately considered that the wall of the liquid metal and the resin are tangents, which simplifies the modeling process.

**Figure 5:**
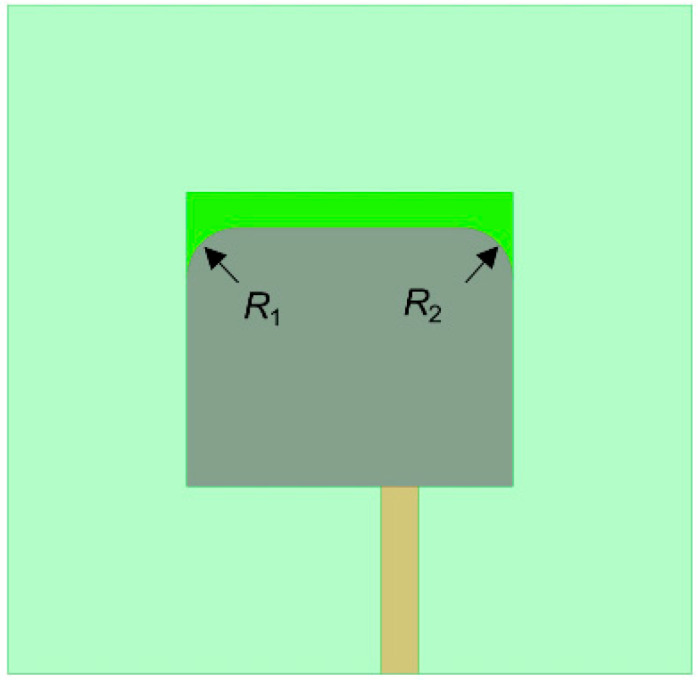
The optimized antenna structure with considering the wetting factor.

Based on the geometric structure of Figure 1a, rounded corners *R*_1_ (at left) and *R*_2_ (at right) are then added to modify the geometric model. The modified model is shown in Figure 5. To improve the accuracy of the models, for different rotation angles, the simulation models are revised according to the sizes of the actual objects *R*_1_ and *R*_2_.

## 5. Rotating Method

**Figure 6:**
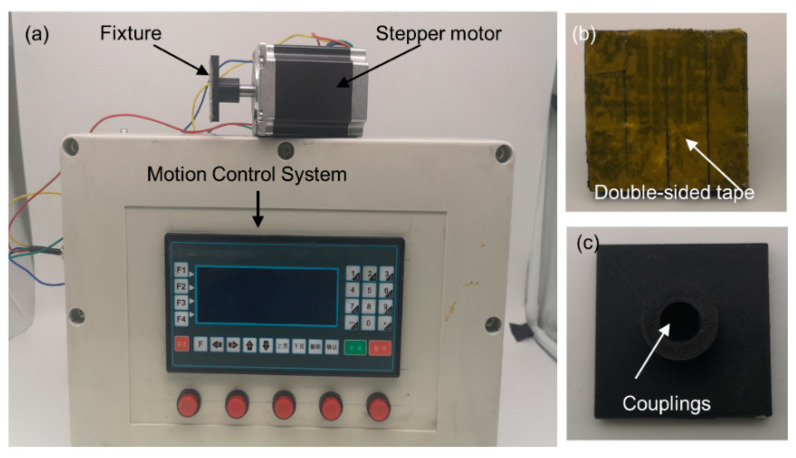
(**a**) The overall view of the turntable. (**b**) Front view of two clamps. (**c**) Back view of the two clamps.

The designed antenna is expected to have different radiation characteristics when the patch plane is rotated at different angles. To realize that, the rotation angle of the antenna could be accurately controlled when measuring the reflection coefficients. In this work, a turntable is designed and manufactured, as shown in Figure 6a which consists of a stepper motor, a motion control system, and a clamp.

The stepper motor is provided by Changlin Automation Technology Co., Ltd., Wuxi, China. The manufacturer model is 57BYGH801, and its rated voltage and current are 4 V and 2A, respectively, and the holding torque is 10,000 g·cm.

The motion control system is produced by Dopcon Automation Technology Co., Ltd., Beijing, China. The manufacturer model is TC55 and it is equipped with a 32-bit central processing unit (CPU), a servo motor, a liquid crystal display, and a contact keyboard. The movement state can be adjusted manually or using a computer. In manual fine-tuning mode the angular resolution is 0.2°, which meets the requirements for accurate angle control.

The clamp is used to fix the antenna and the stepping motor. The clamp can be quickly fabricated using the Fused Deposition Modeling (FDM) 3D printing method. The front and back views of the clamp are shown in Figure 6b,c. The double-sided adhesive on the front of the clamp can be completely attached to the ground plane of the antenna, and the coupling at the back of the clamp connects the antenna with the stepping motor. Another clamp with two fins is also manufactured and used, as shown in Figure S4.

## 6. Results and Discussion

### 6.1. Reflection Coefficient

We use a vector network analyzer (VNA) E5063A (Keysight Technologies, Inc., Santa Rosa, DE, United States) to measure the reflection coefficients of the fabricated antenna.

The turntable rotates the antenna from 0° to −10°, −20°, −30°...−90° in the counterclockwise direction (counterclockwise rotations are marked by “-”). Similarly, the turntable rotates the antenna from 0° to +10°, +20°, +30°... +90° in the clockwise direction (clockwise marked as “+”). The reconstruction process of the antenna at different rotation angles is demonstrated in Movie S1.

**Figure 7:**
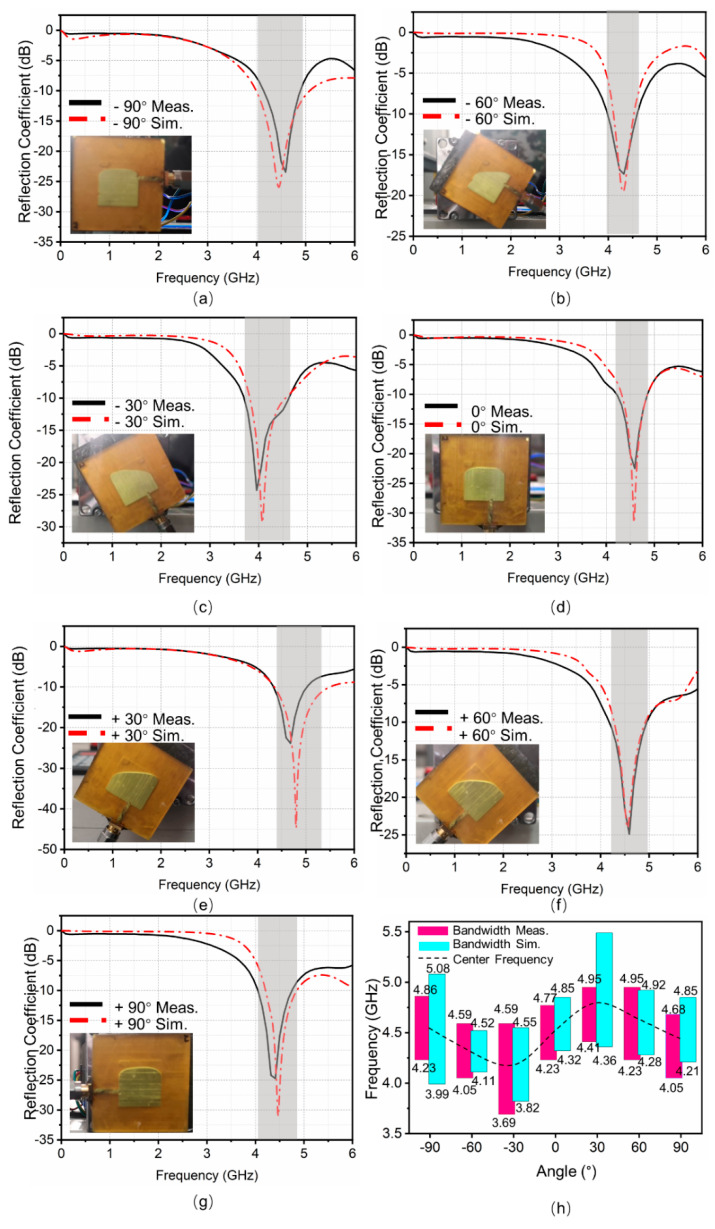
(**a**–**g**) Simulation and measurement results of the reflection coefficients of the antenna for various frequencies, where the rotation angle is increased from −90° to +90° in steps of 30°. (**h**) The obtained impedance bandwidth by simulations and measurements for different rotation angles.

A total of 19 sets of data for 19 angles (see Table S2) are obtained in the measurements. To ensure that results are not redundant, seven sets of data (at −90°, −60°, −30°, 0°, +30°, +60°, +90°) are selected for analysis without affecting the accuracy. The impedance bandwidth and the center frequency under different rotation angles are also presented in Table 2. Figure 7a–g shows the reflection coefficients varied with frequencies at different rotation angles. At −60°, 0°, and +60°, the experimental results well agree with the simulation results. For other rotation angles, there exist some frequency deviations within the allowable error range.

There are many potential reasons for the errors. Firstly, there are several potential causes of manufacturing errors such as the size error of 3D printing and machining, as well as the volume error of the injected liquid metal. In addition, the interface between the liquid metal and NaOH solution in the fabricated antenna is a curved surface. Although the rounded corners are used to correct the model after being affected by the surface tension, there may exist some differences in the model. The main source of error is from manufacturing. Therefore, some high-precision instruments like flaw detectors can be used to scan 3D printed and machined parts to improve the quality of the antenna. In addition, EGaIn can be weighed and used more accurately. Finally, industrial instruments such as 3D scanners can be used to better reconstruct and optimize models.

The antenna is analyzed according to the rectangular patch. In the dominant mode of TM01, the current follows along the length of the patch to realize the frequency reconstruction. To summarize the law of frequency changes clearly, the impedance bandwidths of Figure 7a–g are summarized in Figure 7h. Firstly, the rotation angle is gradually changed clockwise from −90° to −30°, and the resonant frequency decreases. Subsequently, the rotation angle is gradually changed clockwise from −30° to +30°, and the resonant frequency increases. Finally, the rotation angle is gradually changed clockwise from +30° to +90°, and the resonant frequency decreases again. This periodic change is somewhat similar to a sine curve (see Figure 7h). The entire working bandwidth (3.69–4.95 GHz) is measured following the same process.

### 6.2. Radiation Pattern

**Figure 8:**
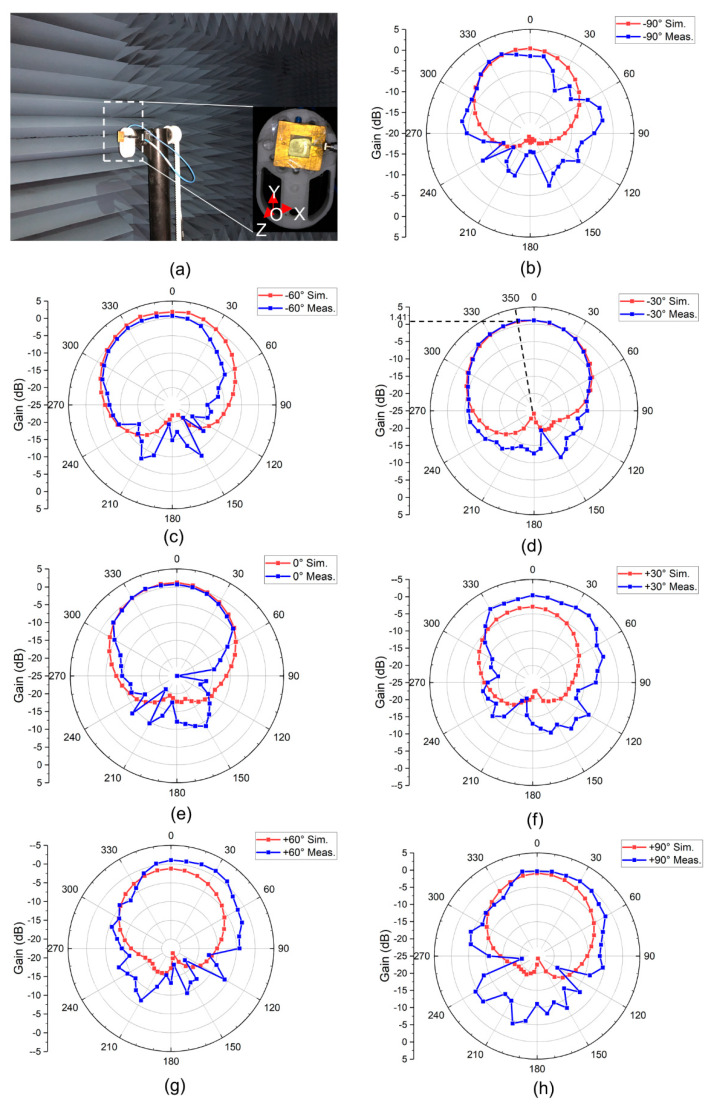
(**a**) The test environment in the microwave anechoic chamber. (**b**–**h**) Simulation and measurement results for the antenna’s E-plane (X-O-Z plane) radiation patterns for various frequencies, where the rotation angle is increased from −90° to +90° in steps of 30°.

The antenna pattern is measured in a microwave anechoic chamber at the Tsinghua University. The measurement environment is shown in Figure 8a. Note that the X-O-Y plane is the plane where the patch antenna is placed. The antenna is rotated around the *z*-axis during the measurements. According to the reference frame defined above, the angle between the microstrip line and the reverse *y*-axis is recorded as the rotation angle. Similarly, for the counterclockwise (clockwise) rotation, the rotation angle is recorded as a negative (positive) value. Therefore, the angle shown in Figure 8a is −90°.

If the patch is placed horizontally (on the X-O-Z plane), the liquid metal would also be deformed in the horizontal plane, which will result in the inability to accurately measure the radiation pattern of the H-plane. Therefore, we only measure the radiation pattern of the antenna of the E-plane when the antenna is placed on the X-O-Y plane. For the same reason, the efficiency of the antenna cannot be measured.

The measurement and simulation results of the radiation pattern of the E plane are shown in Figure 8b–h. The measured radiation pattern is consistent with that of a typical patch antenna, where the main lobe faces the *z*-axis and the sidelobe level is lower, suggesting that the antenna has the end-fire characteristics.

The measurement results are also in agreement with the simulation. The measurements also show that for a rotation angle of −30°, the antenna achieves a maximum radiation gain of 1.43 dBi with the maximum radiation direction of 350°. Except for the manufacturing errors mentioned above, the error sources of the radiation pattern may also come from the measurement environment and measurement process, such as calibration errors and rotation angle errors.

### 6.3. Discussion

For comparison, several frequency reconfigurable antennas based on different principles are presented in Table 3, which includes solid antennas [2,3,4] and liquid antennas [5,6,7,8]. Compared with other works, the antenna in this paper provides a larger tunable range (3.69–4.95 GHz). This operating frequency range covers most of the n77 (3.3–4.2 GHz), n78 (3.3–3.8 GHz), and n79 (4.4–5.0 GHz) channels in the 5th generation (5G) mobile networks. Particularly, for rotation angles within a range of −30° to +30°, the resonant frequency of the antenna is monotonously changed with the rotation angles, which makes it be a promising candidate as a tilt sensor.

However, in many cases, the gain of the antenna is low, i.e., the antenna loss is relatively high. The loss is the collective impact of the liquid metal, NaOH solution, the *HT^®^ 300*, the microstrip feeder, and the manufacturing errors. Firstly, the liquid metal is sealed by the *HT^®^ 300* resin, so the liquid metal patch antenna is covered by the dielectric layer that reduces the gain and efficiency of the antenna. One may consider reducing the thickness of the dielectric layer H3 and its dielectric constant *ε_r_* to further improve the antenna gain and its efficiency. Secondly, it is seen in [24] that reducing the solubility of the alkaline solution improves the efficiency and gain of the antenna. In addition, the ground plane was fabricated using pasted copper foil. If the copper foil is not flat or becomes oxidized during the soldering process, the performance of the antenna would decrease. To address this issue, one can use copper deposition instead.

Although the structure of the rotating-driven liquid metal reconfigurable antenna designed in this paper is simple, as a proof-of-principle design, it provides a new method for the reconfigurable design of liquid metal antennas. The results presented in this paper can be further extended to many different antennas with reconfigurable frequency, pattern, and/or polarization through different patch shapes.

## 7. Conclusions

A frequency reconfigurable microstrip patch antenna based on the principle of liquid metal flowing by rotation was designed, manufactured, and measured. The substrate was easily and quickly obtained by the SLA. The *HT^®^ 300* resin used in the substrate has a temperature resistance of 300 °C. The results of simulation and measurement confirm that the designed antenna shows various radiation characteristics at different rotation angles. The antenna has an ultra-wide working bandwidth of 3.69–4.95 GHz, which cover over range of frequencies suitable for various channels in the 5G mode of mobile communications. The gravity-based reconstruction strategy can be further applied to other liquid antenna designs. Future research works may consider extending the working condition of such antennas to, e.g., horizontal placing.

## Figures and Tables

**Table 1:** Dimensions of the Optimized Antenna (Unit: mm).

*L*_1_	*L*_2_	*L*_3_	*L*_4_
40	17.6	14.6	12.0
*W*_1_	*W*_2_	*W*_3_	*W*_4_
40	19.0	11.5	2.1
*H*_1_	*H*_2_	*H*_3_	*H*_4_
1.0	0.5	1.0	0.1

**Table 2:** The impedance bandwidth and the center frequency for different rotation angles.

Rotation Angles	Impedance Bandwidth (Sim.)	Impedance Bandwidth (Meas.)	Center Frequency (Sim.)	Center Frequency (Meas.)
−90°	3.99–5.08	4.23–4.88	4.44	4.59
−60°	4.11–4.52	4.05–4.59	4.32	4.32
−30°	3.82–4.55	3.69–4.59	4.07	3.96
0°	4.32–4.85	4.23–4.77	4.59	4.59
+30°	4.36–5.49	4.41–4.95	4.80	4.68
+60°	4.05–4.68	4.28–4.92	4.41	4.59
+90°	4.21–4.85	4.05–4.68	4.47	4.41

**Table 3:** Performance comparison for different antenna types.

Ref.	Radiator	Methods of Reconstruction	Reconfigurable Types	Reconfigurable Characteristics	Peak Gain (Mea.)
[2]	Patch	RF-MEMS	Frequency	15.75–16.05 GHz	N/A
[3]	Monopole or Patch	PIN diodes	Frequency and Pattern	2.21–2.79 GHz (Monopole); 5.27–5.56 GHz (Patch)	5.0 dBi
[4]	Patch	Varactor diodes	Frequency	1.92–2.51 GHz	5.91 dBi
[5]	Monopole	Water	Frequency	62.5–180.2 MHz	N/A
[6]	Patch	Liquid crystal	Frequency	2.43–2.53 GHz	0.1 dBi
[7]	Patch	Transformer oil.	Frequency	1.42–1.96 GHz	9.12 dBi
[8]	Patch	Liquid Metal	Frequency and Polarization	5.83 GHz (LP) and 6 GHz (CP) and 6.15 GHz (LP)	3 dBi
This work	Patch	Liquid Metal	Frequency	3.69–4.95 GHz	1.43 dBi

## Supplementary Materials

The supporting materials are available online at https://www.mdpi.com/article/10.3390/mi12060701/s1. Figure S1: *H*_3_ was 0.5 mm, the covering layer of the substrate collapsed; Figure S2: (a) After soldering the copper foil and the SMA connector, the sub-strate made of Accura^®^ 60 deformed. (b) Two substrates made of the Accura^®^ 60 (at left) and HI TEMP^®^ 300 (at right); Figure S3: In an attempted process route, the substrate was made of PDMS; Figure S4. Another clamp with two fins, which are convenient to fix the antenna but also affect the radiation field; Table S1: Performance comparison of the two photosensitive resins; Table S2: The measured full dataset of the return losses of the fabricated an-tenna under 19 angles; Movie S1: The reconstruction process of the antenna at different rotation angles.
