# Supplementary material for: A Gravity-Triggered Liquid Metal Patch Antenna with Reconfigurable Frequency
Source: Micromachines (Basel). 2021 Jun 16;12(6):701. doi: 10.3390/mi12060701 (PMC8234692; doi:10.3390/mi12060701)
Supplement: Supplementary file 1 [file micromachines-12-00701-s001.zip › Supplementary Materials.pdf]

Supporting Information

A Gravity-Triggered Liquid Metal Patch Antenna with Reconfigurable Frequency

Peng Qin [1,2], Guan-Long Huang [3], Jia-Jun Liang [4], Qian-Yu Wang [1,2], Jun-Heng Fu [1,2], Xi-Yu Zhu [5], Tian-Ying Liu [1,2], Lin Gui [1,2], Jing Liu [1,2,5] and Zhong-Shan Deng [1,2,*]

[1] CAS Key Laboratory of Cryogenics, Technical Institute of Physics and Chemistry, Chinese Academy of Sciences, Beijing 100190, China; qinpeng17@mails.ucas.edu.cn (P.Q.); wangqianyu19@mails.ucas.edu.cn (Q.-Y.W.); fujunheng17@mails.ucas.edu.cn (J.-H.F.); liutianying17@mails.ucas.edu.cn (T.-Y.L.); lingui@mail.ipc.ac.cn (L.G.); jliu@mail.ipc.ac.cn (J.L.)

[2] School of Future Technology, University of Chinese Academy of Sciences, Beijing 100049, China

[3] School of AI—Guangdong & Taiwan, Foshan University, Foshan 528225, China; hgl@fosu.edu.cn

[4] School of Physics and Telecommunication Engineering, Yulin Normal University, Yulin 537000, China; shuigpjd@163.com

[5] Department of Biomedical Engineering, School of Medicine, Tsinghua University, Beijing 100084, China; zxy19@mails.tsinghua.edu.cn

* Correspondence: zsdeng@mail.ipc.ac.cn; Tel.: +86-10-82543483

Figure S1. H_3 was 0.5 mm, the covering layer of the substrate collapsed.

Figure S2. (a) After soldering the copper foil and the SMA connector, the substrate made of *Accura*® 60 deformed. (b) Two substrates made of the Accura® 60 (at left) and HI TEMP® 300 (at right).

Table S1. Performance comparison of the two photosensitive resins.

Figure S3. In an attempted process route, the substrate was made of PDMS.

Figure S4. Another clamp with two fins, which are convenient to fix the antenna but also affect the radiation field.

Movie S1. The reconstruction process of the antenna at different rotation angles.

Table S2. The measured full dataset of the return losses of the fabricated antenna under 19 angles.

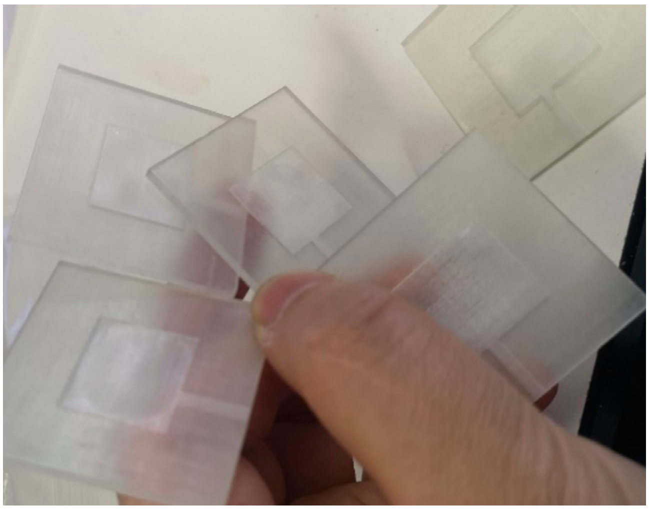

Figure S1. H_3 was 0.5 mm, the covering layer of the substrate collapsed.

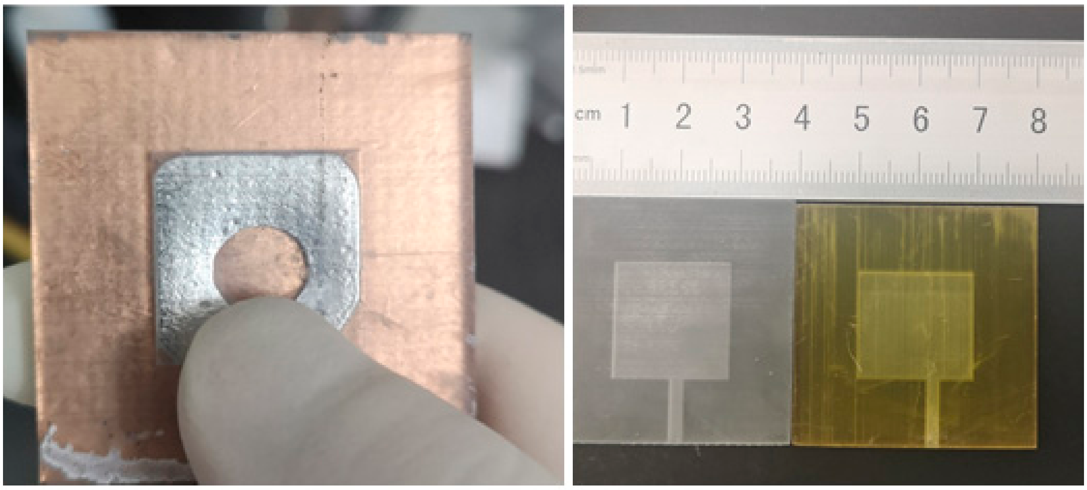

(a) (b)

Figure S2. (a) After soldering the copper foil and the SMA connector, the substrate made of *Accura*® 60 deformed. (b) Two substrates made of the Accura® 60 (at left) and HI TEMP® 300 (at right).

Table S1. Performance comparison of the two photosensitive resins.

The type of material	*Accura® 60*	*HI TEMP® 300*
Printing method	SLA	SLA
The model of the printer	*ProX 800*	*Figure 4*
color	Transparent	Amber
Density in liquid state (g/cm^3)	1.13	1.19
Density in solid state (g/cm^3)	1.21	1.3
Thermal deformation temperature (℃)	53	300

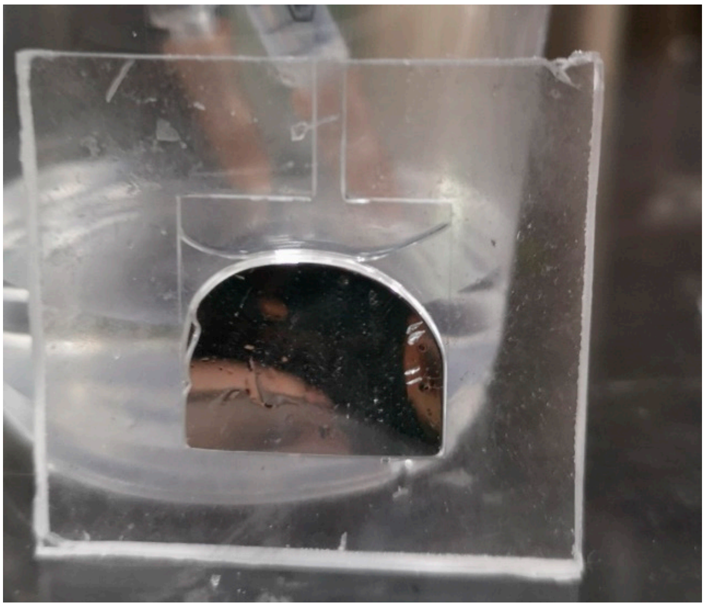

Figure S3. In an attempted process route, the substrate was made of PDMS.

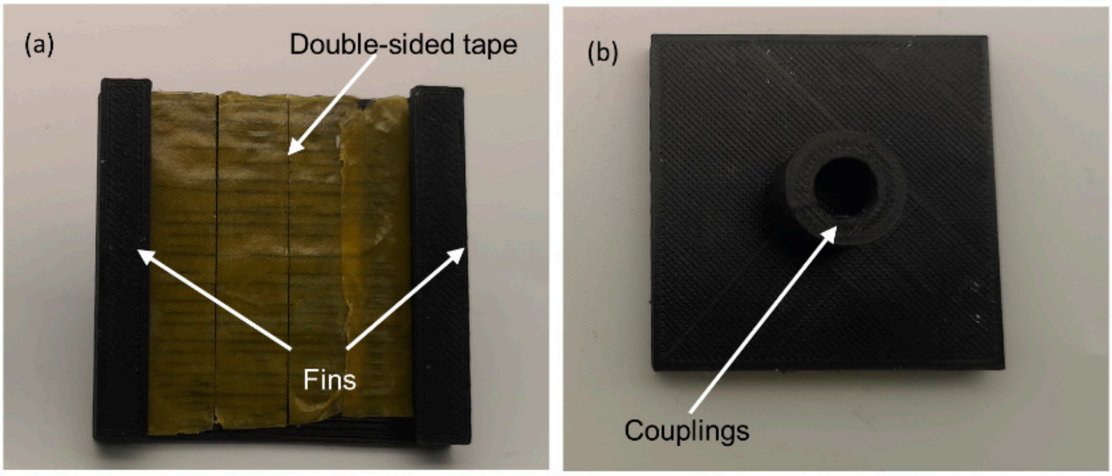

Figure S4. Another clamp with two fins, which are convenient to fix the antenna but also affect the radiation field.
